# Individual attitudes toward innovation, occupational stress and social support for work among HIV health care providers in Southwest China

**DOI:** 10.1186/1748-5908-10-S1-A24

**Published:** 2015-08-20

**Authors:** Shan Qiao, Xiaoming Li, Bonita Stanton, Yuejiao Zhou, Zhiyong Shen, Zhenzhu Tang

**Affiliations:** 1 Prevention Research Center, Department of Pediatrics, School of Medicine, Wayne State University, Detroit, MI 48201, USA; 2 The Center of Disease Control and Prevention in Guangxi, Nanning, China

## Introduction

Existing literature suggests that organizational readiness for change (ORC) can influence implementing and sustaining an intervention program. Individual attitude toward innovation is one of the important components of ORC. There is a growing research effort to identify facilitators and barriers regarding positive attitudes toward innovation in clinical settings. The current study aims to explore how occupational stress and social support for work may affect attitudes toward innovation among health care providers in HIV clinics in China.

## Methods

323 health care providers were recruited from 42 HIV clinics across 12 cities/counties in Guangxi, China to participate in a self-administered survey including measures on demographics, work-related characteristics (e.g., years engaged in HIV-related service, frequency of contacting HIV patients), occupational stress, social support for work (from the family and colleagues), and attitudes toward innovation. We conducted mediation analysis and Sobel testing to examine the relationship among variables of interest.

## Results

After controlling for demographics (e. g. gender, age, race, education level) and work-related characteristics (e.g., working time, training experience), occupational stress was negatively associated with positive attitudes toward innovation (aβ = -0.125, p = 0.04) while higher levels of social support from the family was associated with more positive attitudes toward innovation (aβ = -0.162, p = 0.009). Social support from the family significantly mediated the impact of occupational stress on attitudes toward innovation (Sobel test statistic = 3.0, p = 0.002). However, no significant association was detected between social support from colleagues and attitudes toward innovation (p = 0.600).

## Discussion

Social support for work from the family not only promotes attitudes favoring innovation, but also buffers the negative impact of occupational stress on attitudes toward innovation among health care providers in HIV clinics. Our findings suggest integrating both individual and family level factors into the strategies of improving ORC in HIV clinical settings.

